# Contexts shaping misdemeanor system interventions among people with mental illnesses: qualitative findings from a multi-site system mapping exercise

**DOI:** 10.1186/s40352-023-00219-8

**Published:** 2023-04-04

**Authors:** Jennifer D. Wood, Amy C. Watson, Leah Pope, Amanda Warnock, Veronica Nelson, Nili Gesser, Adria Zern, Aaron Stagoff-Belfort, Jason Tan de Bibiana, Michael T. Compton

**Affiliations:** 1 Department of Criminal Justice, Temple University, Philadelphia, PA USA; 2 Helen Bader School of Social Welfare, University of Wisconsin-Milwaukee, Milwaukee, WI USA; 3 Department of Psychiatry, Vagelos College of Physicians and Surgeons, Columbia University, New York, NY USA; 4 Department of Psychology, University of North Dakota, Grand Forks, ND USA; 5 Vera Institute of Justice, New York, NY USA

**Keywords:** Mental illnesses, Criminal legal system, Misdemeanors, System mapping, Diversion, Criminal charges, Housing, COVID-19

## Abstract

**Background:**

People with mental illnesses are disproportionately entangled in the criminal legal system. Historically, this involvement has resulted from minor offending, often accompanied by misdemeanor charges. In recent years, policymakers have worked to reduce the footprint of the criminal legal system. This paper seeks to better understand how misdemeanor systems intervene in the lives of people with mental illnesses.

**Methods:**

System mapping exercises were conducted with misdemeanor system stakeholders from the jurisdictions of Atlanta, Chicago, Manhattan, and Philadelphia. Narrative detail on decision-making and case processing, both generally and in relation to specific types of behavior, including trespassing, retail theft/shoplifting, and simple assault, were coded and analyzed for thematic patterns. Based on the qualitative analysis, this paper offers a conceptual diagram of contexts shaping misdemeanor system interventions among people with mental illnesses.

**Results:**

All four sites have been engaged in efforts to reduce the use of misdemeanor charges both generally and in relation to people with mental illnesses. Decision-makers across all sites experience contexts that shape how, when, and where they intervene, which are: (1) law and policy environments; (2) location of the behavior; (3) expectations of stakeholders; (4) knowledge of mental illnesses; and (5) access to community resources. Law and policy environments expand or constrain opportunities for diversion. The location of offending is relevant to who has a stake in the behavior, and what demands they have. Clinical, experiential, and system-level knowledge of mental illnesses inform a chain of decisions about what to do. The capacity to address mental health needs is contingent on access to social services, including housing.

**Conclusion:**

People making decisions along the criminal legal continuum are critical to illuminating the dynamic, inter-related contexts that facilitate and frustrate attempts to address defendants’ mental health needs while balancing considerations of public safety. Multi-sector, scenario-based or case study exercises could help identify concrete ways of improving each of the contexts that surround whole-of-system decisions.

## Introduction

Historically, people with serious mental illnesses have been entangled in criminal legal systems (Bonfine et al., 2019; Falconer et al., 2017). Many within this population continuously shift between homelessness, medical institutions, police contact, and jail (Hoge, 2007; Hopper et al., 1997; Isaacs et al., 2019; Samele et al., 2021). Criminal legal interventions with this population come with high fiscal costs to society and are harmful to the people involved in many tangible and intangible ways (Abreu et al., 2017; Wildeman & Wang, 2017). The delivery of mental health services is inherently inadequate in correctional settings, and both short- and long-term detention is a disruptive, isolating, and traumatic experience (Slate et al., 2013). Experiencing police contact can itself be hazardous to one’s mental health (Bowleg et al., 2020).

A person’s entry into the criminal legal system begins with an encounter with police who function as system “gatekeepers” (Neusteter et al., 2019). In this capacity, officers intervene with people who may be living with mental illnesses. Capturing the prevalence of mental health-related police encounters has long been challenging (Huey et al., 2021). By design, police agencies were not established to screen for mental illnesses. However, studies that have employed clinical data sets or self-report data from people with diagnosed mental illnesses have illuminated the nature and extent of police contacts with portions of this population and indicate that despite popular misconceptions, violent criminal behavior does not explain most of the situations that bring people with mental illnesses into contact with police (Morabito & Socia, 2015; Petrila & Swanson, 2010; Swanson, 2018; Swanson et al., 2015). In fact, people with serious mental illnesses are at disproportionate risk of being victims of violent crime (Desmarais et al., 2014; Swanson et al., 2015) and are more likely to take their own lives than to hurt others (Baumann & Teasdale, 2018).

Mental illnesses vary in terms of severity, from mild to serious (National Institute of Mental Health (NIH), 2022), and definitions of serious mental illness (SMI) vary, with some referring to specific diagnoses, such as schizophrenia or major depression, and others emphasizing descriptive criteria such as functional impairment (Gonzales et al., 2022). Hall and colleagues (Hall et al., 2019) examined the arrest records of approximately 600,000 individuals who had been arrested in New York State for either a felony or misdemeanor between January 1^st^, 2010 and December 31^st^, 2013, matching this data with public mental health system records to determine the prevalence of arrestees who had been diagnosed with a major mental illness within 12 months prior to their arrest. They found that 4% to 6% of the sample had been diagnosed with a major mental illness (diagnoses typically considered serious mental illnesses), which compares to 5.6% of the adult population estimated to have SMI in the United States (National Institute of Mental Health (NIH), 2022). Notably, among this sample of arrestees in the study, there was a pattern of “differential adjudication” (p. 1088), whereby a person’s risk of a jail sentence for a misdemeanor offense increased by 50% if they had a diagnosis (Hall et al., 2019). Further analysis of New York State arrest and mental health data (Compton, Zern, et al., 2022) found that most individuals with mental illness indicators had been arrested for Class A misdemeanors. Among 14 Uniform Crime Reporting codes examined, misdemeanor charges of larceny-theft, fraud, and criminal mischief were more common among arrestees with mental illnesses compared to those without.

A recent Georgia-based study (Compton, Graves, et al., 2022) analyzed criminal records of 240 patients enrolled in 3 inpatient psychiatric facilities. Findings indicated that 71% of the sample experienced an arrest at some point in their lifetime. For those who had been arrested, a sub-analysis of their first two arrests revealed common types of charges across the sample, including marijuana possession, DUI, and burglary/shoplifting. Interestingly, a different pattern emerged with respect to the most recent two arrests experienced by people in the sample, where such charges included probation violations, failure to appear, officer obstruction-related charges, and disorderly conduct.

Together, these studies point to a pattern of mostly non-violent and relatively minor misdemeanor offending among people with mental illnesses. What explains their high arrest rate/rate of system contact? Some researchers have focused on police decision-making (Engel & Silver, 2001; Teplin, 1984). One line of research has examined officers’ lack of knowledge and awareness related to mental health symptomology and the levels of mental health stigma police may bring to the job (Compton et al., 2006). Based on concerns that officers lack the requisite skills to recognize mental health symptoms and appropriately respond, substantial resources have been devoted to training law enforcement officers. Arguably the most well-known strategy is the Crisis Intervention Team model that includes 40 h of specialized training for officers. Research indicates the training can improve officer knowledge, attitudes and self-efficacy and increase linkages to care for people experiencing crises (Watson et al., 2017).

Beyond this focus on officer knowledge and capabilities related to people with mental illnesses, other scholars have recognized that the decisions and actions officers take must be understood not only in relation to their own abilities, or the characteristics of the people they encounter, but also in relation to the peculiarities of each situation and community where they police, including access to resources that can assist in providing care (Bittner, 1967). Inspired by the work of police sociologist Egon Bitter (1967) as well as the literature on criminal justice decision-making, Morabito (2007) argues that different “horizons of context” interact to shape the decision to arrest in the course of mental health-related encounters. This includes the “scenic horizon” (community characteristics and known resources), the “temporal horizon” (an officers’ knowledge of a person’s characteristics and history), and the “manipulative horizon” (situational considerations, such as whether a treatment center could swiftly address the needs of the individual).

Yet, police decision-making constitutes only one of the many decision-making stages or “intercepts” (Griffin et al., 2015; Munetz & Griffin, 2006) along the continuum of the criminal legal system. From a system-level perspective that looks beyond a “single-stage” (Mears & Bacon, 2009) there is a need to better understand how misdemeanor systems get, and sometimes stay involved in the lives of people with mental illnesses. To contribute to this understanding, this paper reports on qualitative insights into system functioning that were generated over the course of 4 ‘system mapping’ exercises produced in 4 different jurisdictions in the United States (Atlanta, Chicago, Manhattan, and Philadelphia). The study drew from and adapted a process mapping methodology used to capture case flows and intervention points related to justice-involved populations with behavioral health disorders (Bowser et al., 2018). In each site, participants from criminal legal and behavioral health sectors were presented with draft ‘maps’ of their local misdemeanor system and mental health service activation points. With this visual aid, they helped to refine the maps and to provide explanatory detail on their system processes, decision-making and interventions related to people known or perceived to have mental illnesses. This paper reports on what this narrative information revealed about the common contexts influencing system interventions. The goal of this study was to understand the use and processing of misdemeanor charges in different sites, with the specific aim of identifying shared contexts influencing how such systems intervene in the lives of people with mental illnesses.

## Methodology

This study sought to explain the use and processing of misdemeanor charges, with an emphasis on activation points for mental health-related interventions. It deployed a ‘system mapping’ approach, adapted from an approach used in a NIDA-funded study discussed by Bowser and colleagues (Bowser et al., 2018). Similar approaches, such as ‘process mapping,’ have been used in fields such as criminal justice and healthcare and can help visualize and explain whole-of-system processes, such as the delivery of healthcare services to patients (Arias et al., 2020; Kim et al., 2019; Trebble et al., 2010).

In preparing for the exercise, the research team gathered publicly available reports and documents related to the misdemeanor landscapes in the cities of Philadelphia, Atlanta, Chicago, and New York. Sources included but were not limited to local news media coverage of topics related to mental illness and the criminal legal system, local agency websites describing mental health-related initiatives, and information provided by county, state, or national-level entities working to address the needs of justice-involved populations. Through this preparatory research as well as information provided by study participants, the team learned that all four jurisdictions in this study have taken steps in recent years to reduce the effects of the criminal legal system on people with mental illnesses. Manhattan, Philadelphia, and Chicago are members of the MacArthur Foundation’s Safety and Justice Challenge, making a commitment to implement data-driven solutions to safely reduce jail populations (MacArthur Foundation, 2021). Philadelphia, as well as Fulton and DeKalb Counties (Atlanta), are members of the Stepping Up initiative, a National Association of Counties and Council of State Governments (CSG) Justice Center project, where county elected officials pledge to reduce the number of people with mental illnesses in local jails (CSG Justice Center, 2021).

All four sites have also enacted substantial changes to pretrial detention and bail practices in the past several years. In Philadelphia, as of February 2018, the District Attorney’s office no longer requests cash bail for 25 non-violent crimes (Ouss & Stevenson, 2022; Parent, 2018). That same month, the Atlanta City Council signed a bill that eliminated mandatory cash bail for individuals in the Atlanta City Detention Center (ACDC) for several non-violent misdemeanors (Cook, 2018). In April 2019, New York State passed sweeping reforms to its cash bail systems, making bail requests ineligible for most misdemeanors and non-violent felonies (and thus virtually eliminating pretrial detention), although some provisions of the legislation were rolled back several months later. In February 2021, the state of Illinois passed the Illinois Pre-Trial Fairness Act, a landmark bill that made the state the first in the country to eliminate cash bail payments entirely, although the policy was not planned to go into effect until 2023 (The Crime Report, 2020).

Table 1 provides census data on these sites. Chicago represents the largest jurisdiction. As one of five boroughs in New York City, Manhattan’s population is comparable to Philadelphia, but its median household income is significantly higher. The City of Atlanta is the smallest of the four sites, with the highest population of black or African American residents (51%), followed by Philadelphia (42.1%). Philadelphia’s poverty rate (24.3%) is higher than the other jurisdictions, followed by Atlanta (20.8%).

**Table 1:** Jurisdiction demographics, based on 2019 US Census Bureau data

	**Atlanta**	**Chicago**	**Manhattan**	**Philadelphia**
Population	506,811	2,693,976	1,628,706	1,584,064
Median household income	$59,948	$58,247	$86,553	$45,927
Experiencing poverty	20.8%	18.4%	14.1%	24.3%
People under 65 with a disability	8.7%	7.0%	6.1%	12.7%
White	40.9%	50.0%	64.6%	40.7%
Black or African American	51.0%	29.6%	17.8%	42.1%
American Indian or Alaska Native	0.3%	0.3%	1.2%	0.4%
Native Hawaiian or Pacific Islander	0.0%	0.0%	0.2%	0.0%
Two or more races	2.4%	2.8%	3.4%	3.1%
Hispanic or Latino	4.3%	28.8%	25.6%	14.7%

Based on the literature and site-specific data gathered by members of the research team, the team created a list of common misdemeanor charges among people with mental illnesses. The team also consulted with local stakeholders. Three common charge types were selected across sites that reflect behaviors potentially related to mental health symptomology and social adversities such as housing instability. Those charges were trespassing, retail theft/shoplifting, and simple/misdemeanor assault. Each site also had the opportunity to examine other charges that were of interest. In Atlanta, for example, obstruction of a law enforcement officer and disorderly conduct charges were discussed. Note that even the common offenses selected have unique legal definitions and classifications in each of the four jurisdictions.

The team conducted system mapping exercises through the summer and late fall of 2020 with professionals working in misdemeanor systems and at the interface of criminal legal and behavioral health services in Atlanta, Chicago, Manhattan, and Philadelphia, with approval from each site’s respective Institutional Review Board. Within the study team, specific researchers were responsible for organizing a mapping exercise within a particular site. This included liaising with one or more partner agencies, who had formally agreed to collaborate on the study, to identify people with relevant forms of subject matter expertise. The sample contained people such as decision-makers in different parts of the misdemeanor system (e.g., police, prosecutors, public defenders, judges) and behavioral health sector representatives (from public agencies, or non-profit providers under contract to public agencies) delivering initiatives, programs, and services to benefit justice-involved populations. Court staff included individuals in senior administrative roles and with expertise in specialist courts, respectively. There were also individuals performing roles as analysts and/or agency project coordinators (see Table 2). Using a purposive snowball recruitment approach, each site-based research team relied on recommendations for participants from local stakeholders. In all 4 sites, many, but not all of the participants knew one another by virtue of coordinating or collaborating as part of the case flow process along the misdemeanor system continuum.

**Table 2:** Type and number of participants

Profession / City	Atlanta	Chicago	Manhattan	Philadelphia
Prosecutors	2	5	1	3
Defenders	2	1	2	1
Defense social workers	2		2	
Judges	2		1	1
Court staff			1	1
Police		1	3	1
Behavioral health initiatives and services	5	3	7	6
Analysts and agency project coordinators	1			1
City policy official				1
**Total participants**	14	10	17	15

Prior to the exercise, team members at each site prepared a draft misdemeanor system process map designed to illustrate a typical case flow as well as known points of mental health service activation points. To produce these maps, researchers gathered examples of maps previously produced for the jurisdictions, such as maps produced through the Sequential Intercept Mapping process, or ‘swimlane’ diagrams. A researcher working with the Manhattan-based site built a map using Lucidchart, a web-based application meant for the creation of complex diagrams. This map served as a template for the other 3 sites, where researchers created draft maps representing the unique misdemeanor system structure in each jurisdiction. All draft maps were aimed at visualizing the various pathways a misdemeanor case can take in each jurisdiction and the decision-making components relevant to each path. The draft map was sent to the stakeholders prior to the exercise.

The purpose of developing a draft map was to provide a common visual reference point for discussion, so that participants could focus on providing narrative, explanatory detail on where and how the system intervened in the lives of people that may be living with mental illnesses. Adapting a systems mapping approach used in a study on behavioral health interventions within juvenile justice systems (Bowser et al., 2018), a semi-structured interview protocol was developed with three stages or layers of questions. The first stage was focused on reviewing the draft map and changing it based on stakeholder feedback to accurately reflect the locality’s system pathways. Designated research team members started at the beginning of each map and asked, “What happens next?” beginning with a focal police encounter. In the second stage, team members asked about the decision-making points in the criminal legal case flow related specifically to mental health service activation, such as assessment, referrals, diversions, and treatment. In the third stage, the team asked about the details of decision-making points that may be distinct to the selected misdemeanor charges of interest. Throughout the process, research team members gathered explanatory detail on how cases flowed and the factors that informed decision-making options and choices. These three phases of the exercise were designed to occur sequentially, though facilitators worked to ensure there was an opportunity for organic conversations and narratives to emerge. For each stage, one or more researchers (apart from the lead facilitator) took detailed notes to inform the next iteration of the map. Table 2 contains the number and professions of participants from each site.

The actual mapping exercise for each site was 3 h in duration. Each mapping session was audio-recorded and transcribed, and each research site checked their transcripts for accuracy while listening to the recordings. The transcripts were also de-identified by removing and anonymizing any identifying pieces of information. An initial codebook was developed based on the logic of the exercise which was to understand what was being decided and who was making decisions along the continuum. Codes were organized around the logic of the Sequential Intercept Model whereby, for example, Intercept 1 referred to decisions and decision-makers in and around the police encounter. The first draft of the coding scheme was visualized using Lucidchart as a ‘coding tree’ that moved from Intercept 1 through to the intercept points associated with the post-initial hearing/court stage in the continuum (Intercept 3). This codebook comprised a set of a priori “parent” (or main) codes for each intercept. These parent codes were: *What is being decided?, Who is deciding?, Law and policy factors influencing decision making*, and *Extra-legal factors influencing decision making*. A fifth parent code of C*ommunication* was added to each intercept when it emerged as a theme in each site during the team’s review of the transcripts. The iterative process of codebook development was informed by a collective reading and discussion of excerpts from the transcripts of all four sites. Sub-codes or ‘child codes’ were developed for each of the parent codes and were iteratively modified or merged, such as in instances where unique language is used to refer to the same decision or decision-maker (e.g., ‘site arrest’ in Philadelphia vs. ‘live arrest’ in NYC). Weekly research team meetings facilitated this process. A set of free-floating codes that are independent of the points in the decision-making continuum were also created. These free-floating codes were *mental health context, site history,* and individual *charge codes.*

The study team was responsible for coding and analysis. Once a codebook was finalized, primary coders from each research site (a total of 4 coders) read through their own site’s transcript and applied the codes in the form of a directed content analysis using Dedoose, a qualitative analysis software. To enhance consistency in the coding process across the four sites, the primary coders first all completed a coding exercise, in which everyone coded an excerpt from one of the transcripts. The coders then met to discuss their applications, resolve any differences in coding, and discuss questions that arose. During this coding process, some additional child codes were merged, and the codebook was updated to reflect these changes. Once the coding process was completed, members of the research team generated five main themes around which to organize the findings from the coded data. The themes, which were iteratively refined, represent higher level conceptualizations of the data and therefore encompass material from multiple parent and child codes.

## Results

**Fig. 1:**
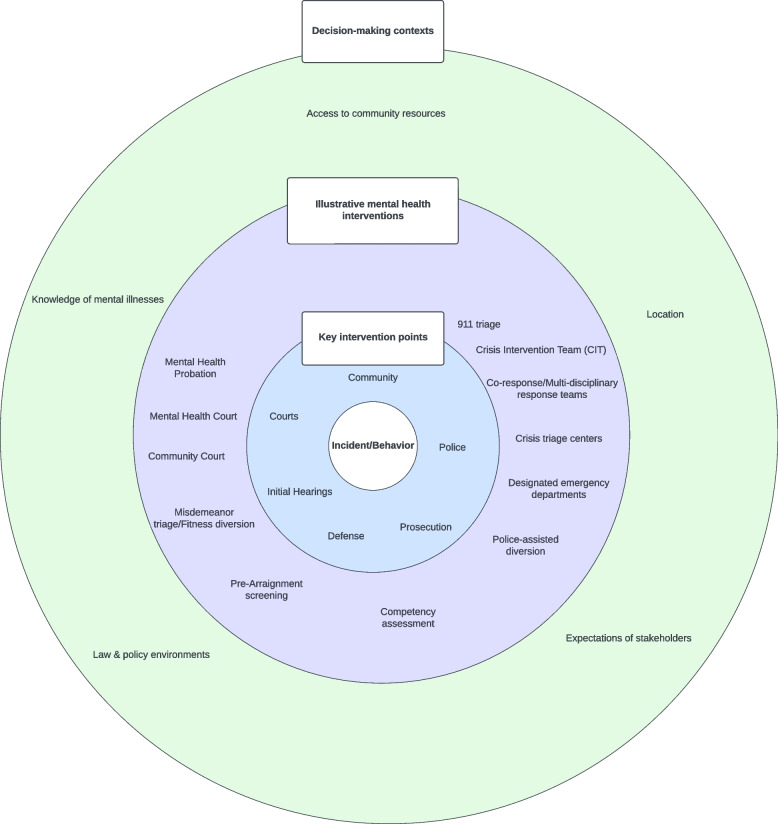
Conceptual diagram of contexts shaping misdemeanor system interventions among people with mental illnesses

The system mapping exercises illuminated the commitment among the four sites to change how misdemeanor systems intervene in the lives of people with mental illnesses. We provide examples of such efforts below. Equally, the exercises yielded rich narrative data on the contexts that influence system decisions and shape what is possible in terms of identifying and addressing mental health needs. A circular (wheel-shaped) visualization of these contexts is displayed in Fig. 1. The Figure depicts a series of intervention points and associated decision-makers, including police, prosecutors, defense, initial hearings, and courts, consistent with the notion of “intercepts” within the Sequential Intercept framework (Griffin et al., 2015; Munetz & Griffin, 2006). Each of the 4 study sites was involved in unique efforts to reduce the involvement of people with mental illnesses in their systems, as captured in the circle depicting illustrative mental health interventions at different points in the system (e.g., crisis triage centers, designated emergency departments, misdemeanor triage/fitness diversion). The outermost circle captures the 5 contexts shaping the interventions of the system as a whole: (1) law and policy environments; (2) location of the behavior; (3) expectations of stakeholders; (4) knowledge of mental illnesses; and (5) access to community resources.

### Efforts to change how misdemeanor systems intervene

All sites shared a commitment to reducing criminal legal system involvement in non-violent misdemeanors, and each of the four sites has initiated unique steps to advance this commitment. For example, beyond existing efforts to address serious mental illnesses through the Mental Health Court, the City of Philadelphia has been expanding its continuum of first response models aided by a new 911 call triage approach and Crisis Intervention Response Team (CIRT) pilot program (Winberg, 2021). With the CIRT program, currently operating in select police divisions, call-takers (as part of the centralized all-city 911 service) identify calls with a mental health crisis component, aided by a mental health script. For certain non-violent call types, a CIRT co-response team—consisting of a police officer and a behavioral health clinician—may be dispatched to respond to the call or may be called to the scene by officers that provided the primary response. Depending on the needs of the individual, a peer specialist follows up to support them in engaging services.^1^

Another program in Philadelphia is its Police-Assisted Diversion (PAD) program—also operating in select police divisions—to divert people with behavioral health-related issues away from the criminal legal system through opting for a treatment versus arrest alternative which provides a pre-booking offramp for people who meet the eligibility criteria. Successful diversion through PAD involves eligibility screening (while under arrest, but prior to booking), approval and diversion to a case manager and community service providers. Treatment is not mandated.

A unique innovation in Chicago is the implementation of mental health triage centers to address the urgent and emergent mental health needs of community members, including those with criminal legal involvement. Chicago Police Department policy directs officers to transport individuals in need of emergency psychiatric care to designated hospital emergency departments or one of three crisis triage centers. Individuals that are under arrest can only be transported to designated emergency departments. Misdemeanor Triage/Fitness Diversion is a deferred prosecution option for individuals with serious mental illnesses presenting with competency issues. Instead of sending the person through a lengthy fitness to stand trial evaluation and competency restoration in preparation for resolving the case, a Triage Court immediately connects participants to services via the Westside Community Triage and Wellness Center. This outpatient’s program length varies (60–90 days) depending on how long it takes for the person to follow through on recommendations. In general, charges are dismissed on completion.

New York City has also worked to improve processes for identifying people with mental health needs at the earliest stages of criminal legal system involvement. Launched in Manhattan in May 2015, the Enhanced Pre-Arraignment Screening Unit screens all people awaiting arraignment in central booking for physical and behavioral health needs (mental health and substance use-related needs). With permission, this information can be shared with defense counsel prior to arraignment. If a defense attorney suspects that their client may not be competent to stand trial, they can request a competency assessment under Criminal Procedure Law (CPL) 730. If the person is deemed incompetent and is facing only misdemeanor charges, they will be transported to a hospital for evaluation under CPL 730 Final Orders of Observation and their charges will be dismissed. After evaluation, they can be civilly committed, released, or can voluntarily remain for treatment.

Like Philadelphia, Atlanta has a Policing Alternatives & Diversion Initiative (PAD, formerly Pre-Arrest Diversion) that allows community members via non-emergency hotlines or police officers to refer individuals who may otherwise be arrested to the program. PAD staff respond on-site at the time of the interaction to address immediate shelter needs, engage the individuals in care, and prevent the current arrest. During the time of the systems mapping exercises, PAD was only available in a couple of community zones, but it is now available citywide.

In Atlanta, the handling of misdemeanors is divided between the State Court system in Fulton County and the Municipal Court system in the City of Atlanta. Restore Atlanta is the Atlanta municipal community court. This is not specifically for those with mental health needs, but mental health treatment can be one component of the court. To enter Restore Atlanta, individuals must plead guilty to the charge. Upon successful completion of the program, the judge can withdraw the plea. If they do not complete the program, they go back to the municipal court for sentencing. Misdemeanor Mental Health Court (Fulton County) is a treatment court program for those with mental health concerns. Individuals can be referred to the mental health court any time after arrest through the trial. The behavioral health teams conduct assessments with individuals to determine treatment and release options.

In short, all 4 sites have engaged in unique efforts to reduce the footprint of the criminal legal system in the lives of people with mental illnesses. Illustrative efforts from across the sites are captured in Fig. 1. Participants’ descriptions and explanations of how their systems function also reveal five common decision-making contexts across the sites. Each of these are discussed next.

### Law and policy environments

The life of a case is influenced by the wider environment in which the original incident took place, including a dynamic law and policy context that influences how decision makers act (Damschroder et al., 2009). Sometimes policy changes occur in response to events that animate public debate about a law and its negative consequences—like fatal police encounters or public debate about institutional racism or the COVID-19 pandemic. In Philadelphia, for example, police policy on how to handle trespassing behaviors was changed following an incident at a Starbucks store in 2018 where police arrested two Black men, which sparked an outcry about how the law was applied (Winberg, 2018). The new policy states that officers must prioritize efforts to explain to the individual(s) that they are on private property and to mediate and deescalate the situation between the person and the property owner (Sacks, 2018; Winberg, 2018). A police representative explained,

…*we have new steps in the police department where a supervisor needs to be called to the scene…and the supervisor’s the one that makes that determination whether or not that person's gonna be arrested for criminal trespassing or not. The store owner or the complainant must go to the detective division of occurrence for that to get to the next level. But 9 out of 10 times, the police officers and the sergeants try to convince the person that's trespassing to leave. We don't want to make an arrest… (Philadelphia)*

We note that following this policy change, the City of Philadelphia effectively decriminalized defiant trespass into a code violation, which made the policy moot.

Philadelphia’s shifting stance on trespassing behaviors took place at the local level, but changes to state law also have a direct impact on local policing. Turning to the example of retail theft/petit larceny, a Manhattan-based participant highlighted that the New York State Bail Reform law has reduced the use of charges and thereby jails for pretrial detention. It has also prompted expansion of Alternatives to Incarceration (ATI) programs. A prosecutor suggested that the bail reform environment likely facilitated the development of this alternative pathway which would reduce the harms of jail and better address the drivers of repeat shoplifting, noting:

*We're hopeful that these people that are getting sentenced to these ATI—they're meeting with the social worker and they're being screen[ed] and they're being offered all kinds of voluntary services—we're hopeful that [it] in the bigger scheme does more good than just giving that person a 60-day jail sentence, they do 40 days in they’re out without any kind of help.* (Manhattan)

Beyond state and local changes to law and policy, the COVID-19 pandemic has had ripple effects on the functioning of criminal legal systems. Across the United States, jurisdictions changed operating policies to address the public health threat of the pandemic by reducing exposure between police and citizens as well as reducing exposure within jail and prison settings (Jackson et al., 2021). A prosecutor from Chicago noted that in the wake of COVID-19, police were issuing tickets instead of making arrests, a shift in policy that allowed (at least for a period) police to have less contact with people with mental illnesses. This prosecutor stated that “during COVID…there was a shift in police interaction with people that allowed them to issue tickets without arresting people, giving them a future date to come to the branch courts.” The prosecutor added that “[i]t's just something [to] keep into consideration that it will be a situation where a seriously mentally ill person will just be…there'll be no one to monitor them at all. Right. They'll just be back out on the street” (Chicago).

Although COVID-related policy helped to reduce jail stays, jails also provide a touchpoint at which behavioral health specialists can screen for potential mental illnesses. An Atlanta-based judge in a misdemeanor Mental Health Court commented on the missed opportunities for mental health interventions in the jail setting, saying,

*… it has somewhat come to a screeching halt because…they're processing you in and processing you out of the jail due to the pandemic before the team can actually get to you… And with the people at the jail screening…the mental health provider agency… the social workers from behavioral health who are at the jail, as well as the public defender and the prosecutor who all have people who are trained to look for them… that is a tremendous safety net that we can utilize hopefully once the pandemic… has subsided… (Atlanta)*

Beyond a dynamic law and policy environment, another dynamic context shaping how misdemeanor systems intervene is geography. Across the four sites, we heard from participants that the location or geography of the behavior is relevant to how the behavior is understood and treated.

### Location of behavior

In recounting how their systems work, participants often discussed prototypical situations, especially in discussions about specific chargeable behaviors like trespass or shoplifting. Location was a theme in their narratives, comprising the place-based context of the behavior and the behavioral norms operating in the place. A defense social worker in Manhattan provided three scenarios where they encounter trespassing charges, and in each scenario, they note the geography of the behavior:

*The only time I ever see a trespass charge these days is something where a person is in a New York City park after closing and they’re charged with trespass instead of the park regulation, also sometimes in shelters, where a person has come in, obviously, supposedly as an invited guest, but not as an invited guest. So, the shelter will ask for trespass charges. And then the third one is sort of like DV [domestic violence]-related where the person is going back, hasn't really violated the Order of Protection, but clearly he's not supposed to be on the premises... (Manhattan)*

Within public settings that are not privately owned (e.g., parks, train stations, bank vestibules), there may be competing understandings of what is acceptable behavior. In Atlanta, for example, a public defender spoke to the challenges of regulating public spaces and intervening when people refuse to leave:

*Criminal trespass for us is pretty much whatever the people in the neighborhood decide it is when they don't want the people who they consider priors or undesirable. We had a participant in our Mental Health Court who was standing at a bus stop near where the store was and in the middle of the program, they arrested him again. And he was at a bus stop…* (Atlanta)

Part of the place-based setting of the behavior is the people or set of stakeholders—including, complainants—that set expectations as to what should be done about the behavior. Beyond the initial complainant, various stakeholders (including the person involved and their families) express views about what should be done at different stages of the criminal process. Taken together, these expectations shape how the criminal legal system intervenes.

### Expectations of stakeholders

Participants from across the sites addressed the role of complainants or victims in shaping decision-making throughout the system. In the previous section on place, an example was provided in Manhattan of shelters asking for trespass charges. Businesses and store owners also enlist the police and build the case. A prosecutor referred to “store managers and loss prevention people that are calling the police to say, 'Please, we arrested this person ourselves, we detained them in our own holding cells, we've taken their photograph… so they don't come back here again, please take them …’” (Manhattan). In Atlanta, a county-level solicitor was discussing uses of disorderly conduct and criminal trespass charges, claiming that criminal trespass is more likely to be a repeat behavior. The solicitor stated, “We'll have defendants who come back to the same location time and time and time again. And so, at that point, the store owner is… reaching out to our office trying to figure out what can be done.”

A Manhattan-based service provider asked a prosecutor how their office deals with requests from stores for orders of protection where someone is repeatedly stealing from them—what they described as the “frequent flyer population.” The response was that stores are advised by the district attorney’s office to leverage their own private authority in banning such people from their stores when the behavior is not violent, which then sets the legal foundation for further punitive action should the behavior not cease:

*… we're not in favor of issuing orders of protection on cases that don't involve violence or threats of violence to our victims…. So when a store has a person who repeatedly steals from them and it's a property crime, it’s not a crime of violence. They're stealing property or merchandise, and they want that person banned and they asked us for an order of protection we generally tell the… store we can't get an order of protection in this case. Nor would a judge sign off on one. But what you can do is you can issue a trespass notice to this person so that way they're not coming back to your store, and if they do come back to your store, they’ll be arrested for trespassing, or if they come back to your store and they steal, they can be arrested for more serious crime like burglary… I think the trespass notice is what most stores are doing and they have their own notices. (Manhattan)*

Community interest groups can also exert preferences about how the criminal legal system should intervene. In discussing disorderly conduct, Atlanta participants noted that the voice of community interest groups can be both powerful and frustrating. A judge stated,

*We have some very powerful people in the community who can raise four dollars’ worth of hell about something, but will not give a penny to try and help come up with resources. They want us to throw them in jail for a disorderly conduct, for a shoplifting and every data that we know says you're gonna [be] spending a gazillion dollars housing them for that, but you won't give us 50 cents on the dollar to put a place for them to get treatment, to get mental health treatment.* (Atlanta)

In some situations, complainants may not wish for charges to be pursued, but rather hope that the person will be provided with services. This can be the case when defendants are known to victims, as in family or intimate partner violence situations. A Philadelphia-based judge noted that families can struggle with the idea of calling the police on a loved one, referring to situations where “family members… agonize over whether or not to call the police when they had issues with family members… they opt not to, and then they subsequently call the police when the person is no longer on site” (Philadelphia).

Stakeholder expectations are heightened, and can vary, when assaultive behavior is at play. Philadelphia participants discussed assaultive behavior and the legal distinctions between simple assault (a misdemeanor) and aggravated assault (a felony). The consensus was that there is a system emphasis on diverting individuals with mental health challenges and charged with simple assault, and in cases of aggravated assault, there is a commitment to lowering the charge to simple assault “[p]rovided”, a prosecutor noted, “that somebody’s going to follow the mental health services” (Philadelphia). During this discussion, a behavioral health professional stated, “Isn’t it just important to note that frequently if there's a victim that they get a say also in this process?” A prosecutor replied,

*Absolutely... And what surprised me in the city when I started talking to complaining witnesses is how sympathetic people can be to the mentally ill and how much mental illness has touched people's lives…They'd like to see the person get the service, the treatment that that person needs. But yes the complaining witnesses have a say when there's a victim involved. Absolutely. (Philadelphia)*

To a limited extent, defendants also have a say in the outcomes of their cases. Public defenders are an essential advocate in this regard, and in some cases, defendants may not wish to avail themselves of mental health services. In discussing how the problem of criminal trespass is handled with mental health considerations in mind, a public defender in Philadelphia noted, “My major concern is obviously my legal duty to my client and keeping them out of jail first off. Secondly if they want services. I mean you know I can't tell you how many times the family says, ‘Hey I want services,’ and my client says, 'I'm fine.' So legally I'm bound to the chagrin of relatives, friends, and family members to abide by what my client wishes.” In discussion of retail theft, it was noted that there may be no opportunity to flag a mental illness, and it even may not be in the interests of the defendant to have this issue flagged. A member of the District Attorney’s Office offered the following reflection:

*as a defender… your job is to get the client the best outcome possible. And there's the question, does that make you say well you have to get the person mental health services? If you can get them a year probation without anything else that they have to do… What’s your job as a defender… to defend that crime? Or kind of commandeer your clients’ choices? (Philadelphia)*

Decision-makers are therefore confronted with different, and sometimes competing sets of expectations when deciding if and how to intervene in cases that involve mental illness. Also informing their decisions is the knowledge they have acquired, either formally or experientially, about a person’s mental health.

### Knowledge of mental illnesses

Knowledge that a person may have a mental illness is acquired in different ways and by different decision-makers including police, defense counsel, prosecutors, correctional staff, and judges. Clinical knowledge is one such form, based on formal evaluations (screening, assessments) carried out by trained behavioral health professionals. Considerations of serious mental illnesses have long been a feature of criminal legal systems in relation to notions of competency or fitness to stand trial. In Chicago, for example, when fitness issues are raised, a Behavioral Clinical Examination would be ordered by the judge. The assessment and fitness restoration process is lengthy. As noted previously, a newer initiative aims to triage people for fitness at the court stage through the West Side Triage Center which is operational in select areas of the city. Rather than pursuing the formal fitness and restoration process for low-level misdemeanor charges, the triage court works to rapidly connect people to services. Once service connections are made, charges can be dropped. A prosecutor explained that once a person arrives at a branch court, “It’s really up to the public defender to bring it to the court’s attention that this person appears to be suffering from a serious mental illness…” (Chicago). Individuals who present with potential mental health problems, but do not present with legal competency issues, can also be evaluated and treated in the Fitness Triage program or via the Deferred Prosecution Program, which results in the successful dismissal of charges if an individual complies with the treatment program. Judges can also activate a mental health assessment. A different prosecutor in Chicago noted, “The judges are also very involved in referrals in a triage. There may be instances where someone hasn't been appointed the public defender and is exhibiting mental health issues in the courtroom itself… It's a very fluid interaction… and …there can be referrals after someone's been put on probation, post disposition and mental health issues arise.” (Chicago).

As noted above, public defenders are essential advocates for their clients. As part of this role, they are oriented toward identifying whether defendants may have a mental illness. In Chicago, the public defender’s office has a process to screen all individuals at Cook County Jail prior to bond hearings when an individual comes in front of a judge for the first time. If a mental health issue is identified, a service plan can be presented to the judge if the client agrees to sharing the information. A mental health professional explained:

*…the public defender's office does these initial screens with folks before they see the judge pre-bond and they flag individuals who appear to have a mental illness. [T]he individuals that we identify, we then follow to determine whether or not they're going to be released if they get an I bond*^2^* or if they're held in custody at Cermak [hospital at Cook County Jail]. And we can follow them either route if they're interested in our services, which are voluntary.* (Chicago)

A representative of the behavioral health sector in Philadelphia claimed that public defenders have a great understanding of and ability to assist clients with mental health issues compared to court-appointed or private counsels:

*The public defender is incredibly knowledgeable about working within the mental health court system and navigating all the next steps that we're about to see. Whereas 9 times out of 10, a court appointed or private attorney does not understand the system. I've heard attorneys say open in court, ‘this is your world, I don't really get it.’* (Philadelphia)

Defense attorneys are not the only personnel responsible for flagging the need for mental health evaluation or treatment. A City Solicitor from Atlanta highlighted the important role of the prosecutor in this regard, noting that not all prosecutors are equal in terms of taking the same initiative:

*I think… another challenge is… identifying when there's a mental health issue. So… I've made the heavy recommendation that… the public defenders are saying, "Oh, well, let's get some treatment." If I'm not making the heavy recommendations or… the prosecutors aren't making those heavy recommendations, then the people are getting time served and they're getting arrested… over and over again… for different offenses. (Atlanta)*

Participants referred not only to clinical knowledge, but also to types of knowledge gained through experience, and experiential knowledge can be bound by geography. A Philadelphia-based officer who works in a part of the city known to have a high concentration of mental health-related situations claimed that police in this district know when to enlist a Crisis Intervention Team (CIT) officer. They stated, “a lot of the officers especially around the area where I work know and they can tell that somebody has… a severe mental problem. And they'll ring the supervisor who in turn the supervisor should, if they're not CIT-trained, summon a CIT officer to that scene” (Philadelphia).

Defense attorneys also refer to patterns across previous cases they have handled. In discussing retail theft, a defense attorney from Atlanta stated, “A lot of times shoplifting is a survival crime. It’s, you know, food…things like that. So… it’s an indicator of homelessness, which is disproportionately usually the mentally ill…” (Atlanta). In their comments on the processing of criminal trespass charges, a Chicago-based prosecutor spoke about how they spot ‘flags’ about mental health:

*… criminal trespass is usually more of a nuisance sort of crime. So the victims aren't necessarily jumping up and down… about it. And usually it gets to the point where they call the police. It's a little bit more of a flag for us because it's…usually somebody… who just refuses to stay away. And I mean, it's you know, we have a whole group of people who march back and forth between the Federal Building and the Daley Center and 26th Street filing things. You know, they're all… clearly mentally ill…. we have a very frequent contact in First Municipal who trespass[es] at ABC all the time because he wants to go and tell his story to the news there. So it's a lot of those kinds of things. … That's usually a little bit easier for us to see as a mental health issue as opposed to retail theft where it could be a mixed bag of motivation. (Chicago)*

A member of the District Attorney’s Office in Philadelphia similarly stated, “[s]omebody's going in there [a place] when more often than not they think they have a right to be there or have no other place to go, but they don't intend to commit a crime therein. That's a flag for them [members of the Charging Unit in the DA’s Office] now to try to alert mental health on this…” (Philadelphia).

Police officers’ knowledge and awareness related to mental illnesses and symptomology shape their decision about how to resolve a situation. An Atlanta public defender noted the relevance of officers’ knowledge to decision-making around the use of obstruction^3^ charges:

*How that charge comes about often, unfortunately, depends a lot on what the officer knows about mental illness and about what they're perceiving from that person, right, especially… in a misdemeanor context. Felonies are different, right, because there's a fight… But, whether or not you see obstruction attached on a misdemeanor offense, often just depends on what the officer knows and, and understands about that interaction that they're havin' with that person… They could walk away with an obstruction or they could walk away with a trespass, right? Or, they could walk away with a disorderly, all because, you know, someone who might be symptomatic for one reason or another doesn't wanna be grabbed. And that officer walks up, puts a hand on an elbow and then the person reacts… They're very frustrating to see because they're often just manifestations of somebody's diagnosis when you see mental illness and obstruction…*

Beyond knowledge that an individual decision-maker may or may not possess, system-wide knowledge might be lacking due to gaps in the passing of information from one decision-maker to the next. For instance, in Philadelphia, it was noted that if signs of mental illness are not captured in a police report, the District Attorney’s Office (in particular their Charging Unit^4^) may not immediately be aware of the need. A member of the District Attorney’s Office stated, “People who present as mentally ill… if it’s not flagged for us in the police paperwork and we can’t sort of tell by the charges, we’re just sort of going by our guts. There’s no additional screening that we receive as to that unless it’s a part of the case received” (Philadelphia). The Philadelphia Police representative commented on the absence of space on police forms to note suspected mental illness: “I’ve never seen anything that would suggest that… any [police] paperwork that would say that this person is 302 [meeting criteria for an involuntary evaluation]. If whatever supervisor responded to the scene and decided ‘we’re just gonna arrest him’ you know… ‘we’re not gonna 302 him…’… I’ve never seen any box checkbox that said possible 302 or 302 tendencies” (Philadelphia).

Having access to community-based behavioral health and social services, including housing, is obviously vital to address defendants’ needs, assuming those needs are known. Such resources must be available, responsive to people’s unique challenges, and accessible, either geographically or in terms of eligibility criteria.

### Access to community resources

Access to community resources is constrained in part by geography and jurisdiction. When asked about options for people who lived outside of the specific areas of Chicago that deliver triage services, a prosecutor noted, “That's been a problem, frankly. So we've gotten around it by transferring cases to where the triage programs are located. We've only done that in a couple of instances, though, the fact that it's just serving a certain part of the city… we're working on expanding that now*.*”

One’s access to pre-arrest diversion options also may be contingent on the type of arrest: a site arrest (known as a “live arrest” in New York) versus a warrant arrest. In Philadelphia, when an officer establishes probable cause on scene, they can conduct a site arrest and detain the individual at the relevant police district. They can also choose not to detain a person and later submit an arrest warrant. In the case of misdemeanors (in contrast to felonies), if an officer does not observe the alleged behavior, then an arrest warrant is often the only possible legal action.^5^ It was noted by Philadelphia-based participants that while warrant arrests are “the exception rather than the rule,” as explained by a member of the District Attorney’s Office, being arrested via warrant closes off opportunities for pre-arrest diversion. They explained, “[O]nce the warrant is signed, one of the things that happens is that… we do draft the criminal complaint at the time of the approval of the warrant, and that complaint is then locked. So… we’re not in a position on warrant arrests to be able to do a pre-arrest diversion because the warrant’s already been approved and signed by a judge or magistrate.”

Access to resources can also come down to logistical issues, like transportation. A public defender from Atlanta spoke of the challenge of the Sheriff’s office to transport people with mental illnesses from jail to treatment facilities after the sentencing process. They explained that Sheriffs have formal responsibility to carry out this transport because people are in their care until such individuals arrive at the facility and the sentence is commuted. They stated that “when our clients are labeled as combative, and our SMI's often are, it takes two people to transport…” As they continued to discuss this issue, the defender added, “I'm getting two or three requests every day for transports. I can only do one a day. So I'm gonna be paying… The jail is now paying for $92 a day, for the county, for people to stay a week longer, 'cause we can't transport them.” Another defender noted that the COVID-19 pandemic is aggravating this issue, stating, “[a]nd then our clients are losing their beds. So if you lose your bed this week, you may not get another bed for three weeks. So it's not like a day or two, it could be… Especially with COVID now, I mean, we just had another program tell us this week, "They're not taking new people." So if you missed your bed there, you're the Sheriff's Department’s bill for a while.” As the discussion continued, it was noted that in this situation where a sentence is commuted for time served when people enter treatment, the person in question does not have the option to leave custody and wait for an available bed. This can mean a protracted period of waiting in jail—up to several weeks and more. Yet, it was noted that judges do worry about sending the individuals back onto the streets, and if an individual does not want to wait for a bed, they may end up serving their time in jail, as one defender said:

*[M]ost of my clients are willing to wait in jail for a bed space in that program. And those that don't and get really antsy and demand to go in front of the judge, get three years in prison. My client... That just happened to a couple clients of ours, in front of a judge, um, they got tired of waiting with COVID and they wanted to see the judge. And the judge just gave them the whole time. (Atlanta)*

Access (or not) to mental health resources can thus shape the nature and degree of misdemeanor system entanglement for people with mental illnesses.

## Discussion

Participants’ narratives from the multi-site system mapping process illuminated different contexts shaping how, where and when misdemeanor systems intervene with people with mental illnesses. These contexts are dynamic and work together to influence the full spectrum of system interventions. A circular (wheel-shaped) visualization of these contexts is displayed in Fig. 1. Our focus here has been primarily on the outer circle of decision-making contexts that influence intervention decisions in different ways and at different points around the circle: (1) law and policy environments; (2) location of the behavior; (3) expectations of stakeholders; (4) knowledge of mental illnesses; and (5) access to community resources.

Law and policy environments provide legal tools to regulate behavior, and such environments can and do change, such as when Philadelphia effectively decriminalized the charge of defiant trespass, or when New York State – through its bail reform – reduced the use of charges leading to pre-trial detention. The location of behavior influences how police are mobilized to intervene. Community members may enlist the police to address the undesirable behavior of a Mental Health Court participant at a bus stop. A store may call the police to address a case of shoplifting. Depending on the location of the behavior, prosecutors may pursue different policies, asking businesses to address the behaviors with their own tools, such as trespassing notices. Surrounding every incident is a set of stakeholders that exerts influences on how the incident is handled, ranging from businesses who ask for orders of protection to keep shoplifters away, to assault victims who want to see an assailant receive mental health services. Such expectations can influence the decisions of not only police, but other decision-makers such as prosecutors and defenders. Knowledge of mental illness also influences how the entire system intervenes. If a police officer is unaware of mental health issues during an arrest, the prosecution may in turn lack knowledge to inform their charging determination.

Just as Morabito pointed to the “horizons of context” shaping police decision-making, the findings point to horizons of context shaping the decision-making of the system overall. From this system view, study findings connect with insights from General Systems Theory (GST) (Bernard et al., 2005) which centers on the study of interconnections among systems and accounts for ‘open systems’ that interact with their environments. From this perspective, ‘system’ is defined as a group of interacting, interdependent elements that form a complex whole. Systems tend to be embedded in larger systems and are perpetually adapting. Bernard and colleagues (Bernard et al., 2005) argue that GST can be fruitfully applied to the criminal legal system. They indicate that the system can be considered as “loosely coupled” as it is comprised of multiple, autonomous bureaucracies with low levels of interdependency with each other. Bishop and colleagues (Bishop et al., 2010) argue that the more decision-makers involved, the more “loosely coupled” a system will be.

From a systems perspective, our findings highlight the value of bringing together criminal legal decision-makers to produce a granular understanding of the contexts shaping how they intervene in the lives of people with mental illnesses. With a shared understanding of each context, decision-makers could identify opportunities for innovation. For instance, system stakeholders can examine the location of a behavior and how the police may or may not intervene, such as when a homeless shelter calls police to remove an unwanted guest, or when a citizen calls police to complain about an unwanted person in a public park. Through a shared examination of the relevance of location, criminal legal decision-makers may identify creative opportunities for place-based intervention alternatives that might enlist non-police resources that are more responsive to mental health-related needs. This might include the use of peers, clinicians or social workers as alternative first responders who work to minimize criminal legal involvement (Watson et al., 2019). Such alternatives are the focus of growing interest among researchers and policy-makers (Beck et al., 2020). As part of this place-based focus, decision-makers might also examine the shared challenges of managing the expectations of different stakeholders including complainants. Retailers might want people removed, or people at a bus stop might associate the symptoms of mental illness with the risk of danger. By examining the expectations of stakeholders in depth, decision-makers may identify opportunities for educating the public about mental health stigma and how to address concerning behavior with care and compassion.

System stakeholders can also jointly examine the ways in which their shared law and policy environments either facilitate or obstruct efforts to best respond to the needs of people with mental illnesses. For instance, bail reform initiatives might help to minimize the use of jail detention but may also reduce opportunities to screen for the mental health needs of defendants. By examining this policy issue in detail, stakeholders may identify opportunities to address mental health needs at an earlier intervention point (e.g., point of police contact), which might open opportunities for offering mental health services. Stakeholders can also examine the ways in which system knowledge of mental illnesses both facilitates and constrains how the system intervenes. As illustrated in our findings, various decision-makers draw from distinct forms of experiential and clinical knowledge in deciding how to address a behavior or process a case. Together, these stakeholders could develop a shared understanding of the forms of mental health-related knowledge that animate their decisions and identify knowledge gaps or mechanisms for sharing knowledge across decision-makers. Such decision-makers could also work together to advance a shared understanding of community resources, including lack of resources and the challenges of accessing resources. Through a collaborative examination of resource challenges, these decision-makers might identify concrete mechanisms for improving resource access, such as expanding transportation options, or collectively advocating for resources, such as triage centers, to be provided in areas that do not have them.

Scenario-based, or case-based exercises might provide a concrete mechanism for criminal legal decision-makers to examine their shared decision-making contexts in depth, and to develop innovative ideas for improving those contexts. Such exercises might follow a modified version of the ‘Sentinel Event Review’ (SER) process which focuses on improving how whole systems function. The SER process was designed to diagnose system ‘errors’ or what Hollway and colleagues refer to as “any undesirable outcome in the criminal process” (Hollway & Grunwald, 2019, p. 707). SERs traditionally focus on a case-based analysis of a ‘sentinel event’ where a significant error or harm occurred, or an event that was a ‘near miss’ took place. It is concerned with thoroughly diagnosing the root cause or causes of an error and identifying practical points of intervention to reduce the chances of the error happening in the future. The assumption is that errors can in some cases be caused by a series of small ‘mistakes’ that combine to produce a big mistake, or even a disaster (Doyle, 2010; see also Sherman, 2018). The approach has been used in a range of industries such as aviation and medicine (Liang, 2000), and the case has been made to apply such reviews to policing and criminal justice (Doyle, 2019; Sheil et al., 2016), as has been done previously in Philadelphia (Hollway & Grunwald, 2019) outside of a mental health context.

An SER approach to diagnosing the root causes of system involvement among people with mental illnesses could be useful in preventing disastrous outcomes like a person with mental illness dying in custody or becoming more ill while navigating the requirements of a diversion program. But it might also be used in diagnosing frequent and prototypical cases that lead to repeat contacts with police or the system. The diagnostic process could center on an analysis of each of the five contexts examined in this study, and how each shapes the system decisions related to the scenario in question. Scenario-based exercises could help participants figure out concrete ways to ameliorate these contexts (laws, policies, flows of knowledge, access to resources, etc.) that improve the quality of interventions by misdemeanor systems, or expand intervention alternatives that do not rely on the tools of the criminal law. Into the future, it is also important to integrate this conceptual focus on system decision-making contexts with the literature on ‘focal concerns’ (Bishop et al., 2010; Ericson & Eckberg, 2015; Ishoy & Dabney, 2018), including the socio-legal contexts shaping decision-making (Lynch, 2019) as well as the principles and normative orientations guiding decisions at different stages of the criminal legal system. To this end, an article (Pope et al., 2023) reports on additional qualitative data collected from the 4 sites examined here that engage with the focal concerns framework and illustrate the ‘competing concerns’ that different decision-makers bring to bear across their loosely coupled systems. Scenario-based exercises like the ones suggested here could include the aim of identifying how different value orientations can be aligned across the system toward better outcomes for people with mental illnesses.

This study was limited by its sample and length of the systems mapping exercises. Across all sites there was not full representation of criminal legal and behavioral health stakeholders, and in one site, a police representative was not able to attend. The framework of the 5 decision-making contexts presented here is the beginning of a framework for understanding misdemeanor system interventions. Most critically, this study did not involve participation from people with lived experiences because it was focused on criminal legal decision-making. That said, future research on misdemeanor system interventions should center community engagement and in particular engagement of people with lived experiences of mental illnesses (Beck et al., 2022; The Front End Project, 2021) in diagnosing system weaknesses and advancing equity in whole-of-system decision-making (Neylon, 2021; Policy Research Associates, 2021). It should also be acknowledged that people with mental illnesses become entangled in *civil* legal systems through mechanisms like involuntary commitment petitions that routinely involve the police (Huey et al., 2021). Moreover, police are deployed by concerned citizens or health professionals who need assistance with wellness checks or in responding to people who abscond from hospitals (Huey et al., 2021), and police are professionals with the legal authority to take people into custody for emergency psychiatric evaluation. In this way, police are ‘gatekeepers’ to different ‘systems’ in their communities, and more needs to be learned about how such systems collectively intervene in the lives of people with mental illnesses.

Finally, an issue that merits much greater attention, and is not captured in our conceptual diagram, is the intersections of gender, race, and poverty in the decision-making contexts of misdemeanor system interventions. Future research should fully identify the ways in which structural racism and mental health inequities (Bailey et al., 2017; Huynh, 2022; Vinson & Dennis, 2021; Vinson et al., 2020) permeate the five decision-making contexts identified here – location, expectation of stakeholders, law and policy environments, knowledge of mental illnesses, and access to community resources – and potentially other contexts that this study did not discover.

## Data Availability

Raw group interview data from the systems mapping exercises are not publicly available to protect participants’ privacy.

## Footnotes

1 Relatedly, a recent option at the point of initial contact emerged in Chicago since the mapping exercise. Comprised of an officer, a clinician and a paramedic, the Crisis Assistance Response and Engagement (CARE) team is being piloted in two police districts as a first response to some behavioral health related 911 calls.

2 An I-Bond is a personal recognizance bond. The person is released after they sign a statement indicating they will come to court. Failure to appear in court on the specified date could result in a warrant and the person having to pay the bond amount.

3 O.C.G.A. 16–10-24 defines obstruction as “a person who knowingly and willfully obstructs or hinders any law enforcement officer in the lawful discharge of his official duties is guilty of a misdemeanor.” https://law.justia.com/codes/georgia/2010/title-16/chapter-10/article-2/16-10-24

4 The Charging Unit reviews all site arrests and arrest warrants and determines whether the District Attorney’s Office will prosecute various charges.

5 There are statutory exceptions where officers can effectuate warrantless arrests for misdemeanors for behaviors occurring outside of their presence, such as simple assault committed against a family member or theft charges.
